# Circulating Free miRNAs as Liquid Biopsy Biomarkers for Early Detection of Breast Cancer in Peruvian Women

**DOI:** 10.3390/cancers18121883

**Published:** 2026-06-09

**Authors:** Diana J. Arenas Machaca, Álvaro Martín de Bernardo, Daniel Alejandro Desposorio-Vicente, Sandro Casavilca-Zambrano, Karen Yoshira Cruz-Hualpa, Bijaya Milagros García-Gómez, Tatiana Vidaurre, Yasser Sullcahuaman-Allende, Juan Jose Contreras-Mancilla, Ruddy Liendo-Picoaga, Gustavo A. Sandoval, Marta Dueñas Porto

**Affiliations:** 1 Unidad Funcional de Genética y Biología Molecular, Instituto Nacional de Enfermedades Neoplásicas (INEN), Lima 15038, Peru; ysullcahuaman@inen.sld.pe; 2 Master’s Program in Molecular Biology, Postgraduate Unit, Faculty of Biological Sciences, National University of San Marcos (UNMSM), Lima 15081, Peru; 3 Institute of Biomedical Research, Hospital Universitario 12 de Octubre (IMAS12), 28041 Madrid, Spain; alvaro.martindebernardo@ciemat.es; 4 Molecular and Translational Oncology Division, Centro de Investigaciones Energéticas, Medioambientales y Tecnológicas (CIEMAT), 28040 Madrid, Spain; 5 Centro de Investigación Biomédica en Red de Cáncer (CIBERONC), 28029 Madrid, Spain; 6 Grupo de Investigación de Bioquímica y Biología Sintética-UNFV (GIBBS-UNFV), Laboratorio de Bioquímica y Biología Molecular, Facultad de Ciencias Naturales y Matemática, Universidad Nacional Federico Villarreal (UNFV), Lima 15007, Peru; 2019004436@unfv.edu.pe; 7 Unidad Funcional de Gestión del Banco Nacional de Tumores, Instituto Nacional de Enfermedades Neoplásicas (INEN), Lima 15038, Peru; scasavilcazambrano@gmail.com (S.C.-Z.); cmjuanjo.contreras@gmail.com (J.J.C.-M.); rliendo@cientifica.edu.pe (R.L.-P.); 8 International Joint Laboratory of Molecular Anthropological Oncology (LOAM), Instituto Nacional de Enfermedades Neoplásicas (INEN), IRD, Lima 15038, Peru; 9 Departamento de Mamas y Tejidos Blandos, Instituto Nacional de Enfermedades Neoplásicas (INEN), Lima 15038, Peru; yoshiracruz@live.com; 10 Graduate Program in Bioinformatics and Omics Sciences, Postgraduate Unit, Faculty of Biological Sciences, National University of San Marcos (UNMSM), Lima 15081, Peru; bijaya.garcia@unmsm.edu.pe; 11 Departamento de Oncología Médica, Instituto Nacional de Enfermedades Neoplásicas (INEN), Lima 15038, Peru; tatiana.vidaurre@gmail.com; 12 Centro de Investigación en Bioingeniería, Universidad de Ingenieria y Tecnología (UTEC), Lima 15063, Peru; 13 Laboratorio de Investigación en Cultivo Celular e Inmunología, Universidad Científica del Sur, Villa El Salvador, Lima 15067, Peru; 14 Research Group in Bioinformatics and Structural Biology, Faculty of Biological Sciences, National University of San Marcos (UNMSM), Lima 15081, Peru; gsandovalp@unmsm.edu.pe

**Keywords:** breast cancer, miRNAs, biomarkers, liquid biopsy, qRT-PCR

## Abstract

**Background**: Breast cancer is the most common cancer in women both in Peru and worldwide. Although mammography remains the standard for early detection, its effectiveness may be limited by unequal access to this technology. In this context, liquid biopsy emerges as a minimally invasive complementary technique that allows the identification of circulating biomarkers, such as microRNAs (miRNAs), whose differential expression has been associated with breast cancer. **Methods**: The present study evaluated the diagnostic potential of cell-free circulating miRNAs for the early detection of breast cancer. In the screening phase, seven candidate miRNAs were quantified by qPCR in plasma from patients with early-stage breast cancer and healthy controls. In the validation phase, miR-191, miR-182, miR-335, and miR-125b were analyzed in an independent cohort of 30 untreated patients to evaluate their diagnostic performance. **Results**: miR-125b and miR-335 showed the best individual diagnostic performance, with AUCs of 0.81 and 0.78, respectively, and presented a significant moderate correlation (ρ = 0.608; *p* < 0.05), supporting their biological consistency and potential as complementary biomarkers. The multivariable binary logistic regression model that integrated both miRNAs showed a moderate improvement in discriminatory ability; however, the expanded multivariable model that incorporated the four validated miRNAs achieved an AUC of 0.91, with a sensitivity of 92% and a specificity of 90%. **Conclusions**: The panel composed of miR-191, miR-182, miR-335, and miR-125b represents a promising set of circulating biomarkers for the early detection of breast cancer.

## Simple Summary

Breast cancer remains one of the leading causes of cancer-related death among women, and early detection is essential to improve patient outcomes. Although mammography is the standard screening method, access to this technology remains unequal in many regions, including Peru. Liquid biopsy offers a minimally invasive alternative that can detect circulating genetic material, such as microRNAs, through a simple blood sample. In this study, we evaluated circulating microRNAs as potential biomarkers for the early detection of breast cancer in Peruvian women. We identified a panel of four microRNAs that showed promising diagnostic performance in distinguishing patients with early-stage breast cancer from healthy controls. These findings provide initial evidence supporting the potential use of circulating microRNAs as complementary tools for early detection in a population that remains underrepresented in biomarker research. Further studies in larger and independent cohorts are needed to confirm their clinical utility and broader applicability.

## 1. Introduction

Breast cancer (BC) is the most common cancer in women worldwide and represents a priority public health challenge due to its high incidence and mortality [1]. In Latin America, its incidence has been increasing in recent decades, with notable variations in mortality between countries [2,3]. In Peru, breast cancer is the most common neoplasm in women [4], characterized by diagnosis mainly in advanced stages [5], a situation aggravated by marked regional inequality in access to mammography, especially in rural and high Andean areas, where the availability of equipment and specialized personnel is limited, contributing to a low survival rate [6].

Breast cancer is influenced by a combination of factors such as lifestyle factors (sedentary lifestyle, unhealthy diet), reproductive factors (late pregnancy, low parity), genetic factors (family history), hormonal factors (physiological changes, therapies), environmental factors (radiation), metabolic factors (obesity) and age [7]. It is characterized as a heterogeneous disease due to its complex spectrum of underlying genetic and epigenetic alterations that determine tumor progression, making each case a challenge in choosing the most appropriate therapeutic approach depending on the specific type of cancer [8].

Breast cancer classification follows the TNM staging system, which assesses the size and extent of the tumor (T), lymph node involvement (N) and the presence of metastasis (M), thereby defining stages 0 through IV [9]. Immunohistochemical analysis complement this staging by identifying key tumor characteristics, estrogen receptor (ER) and progesterone receptor (PR) status, human epidermal growth factor receptor 2 (HER2) expression, and the Ki-67 proliferation index. These markers enabled the classification into four intrinsic molecular subtypes: Luminal A, Luminal B, HER2, and Triple-Negative [10], facilitating personalized therapeutic approaches. For instance, ER- and PR-positive tumors, may benefit from hormone therapies, while HER2-positive tumors may respond to targeted therapies [11].

Furthermore, advances at the transcriptomic level have highlighted the relevance of molecules such as miRNAs as diagnostic and prognostic markers in multiple types of cancer, including breast cancer [12,13]. miRNAs can regulate gene expression at the post-transcriptional level, allowing them to influence key processes such as cell proliferation, apoptosis, and metastasis [14]. In breast cancer, alterations in the expression of certain miRNAs have been linked to increased tumor aggressiveness, resistance to treatment and a poorer prognosis, as they can act as tumor suppressors or promoters, depending on the specific molecular and cellular context, making them potential tools for improving treatment classification and personalization [15,16].

Although mammography is the gold standard method for early detection of breast cancer, its effectiveness is compromised by its low sensitivity in women with dense breast tissue, showing a false negative rate of 15 to 25% [17]. Furthermore, the low availability of mammography machines in Peru is insufficient to cover the entire female population [18]. These limitations highlight the need to develop complementary, minimally invasive tools with high diagnostic accuracy to strengthen early detection. In this context, liquid biopsy allows the identification of circulating biomarkers with differential expression, including miRNAs, which could improve diagnostic capacity and population coverage [19]. Circulating miRNAs have emerged as promising non-invasive biomarkers due to their stability in biological fluids and their involvement in multiple cancer-related processes. Recent studies have highlighted the diagnostic and prognostic potential of circulating miRNAs and extracellular vesicle-associated biomarkers in breast cancer and other malignancies [20,21].

The present study analyses the differential expression of circulating cell-free miRNAs in plasma. The evaluation was carried out in two cohorts using a case–control study design that included patients with early-stage breast cancer treated at the National Institute of Neoplastic Diseases (INEN) and healthy controls. The miRNA expression profile was determined by qRT-PCR, a technique known for its high sensitivity and specificity in nucleic acid quantification [22].

## 2. Materials and Methods

### 2.1. Systematic Literature Review

Before initiating data collection and experimental analyses, a systematic literature review was conducted in accordance with the PRISMA 2020 guidelines [23] to identify miRNAs previously investigated as potential biomarkers for the early diagnosis of breast cancer. Studies published between 2010 and 2024 were retrieved from the PubMed and SciELO databases using the following search terms: breast cancer, plasma, serum, early detection, microRNAs, miRNA, circulating miRNAs, as well as specific miRNAs reported in the literature (e.g., miR-191).

Eligible studies met the following inclusion criteria: (i) a minimum sample size of 10 participants per group (controls and cases), (ii) analysis of tissue, plasma, or serum samples, and (iii) provision of experimental data on the differential expression of miRNAs. Through this process, 15 miRNAs were identified as potential circulating biomarkers, in addition to two endogenous miRNAs commonly used as internal controls in breast cancer expression studies.

### 2.2. Study Design

**Figure 1:**
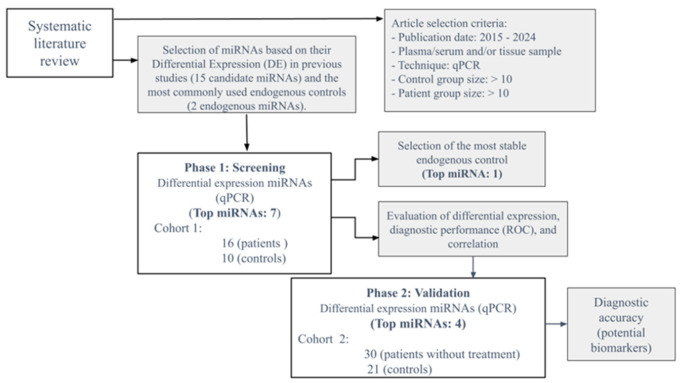
Study Design. (DE: Differentially Expressed).

The study followed a case–control design, consisting of two independent cohorts: a screening cohort (*n* = 26) and a validation cohort (*n* = 51) (Figure 1). Both cohorts included patients with a histological diagnosis of early-stage breast cancer (stage 0, I, and II) and a control group of healthy women with no personal history of cancer.

The patients were diagnosed and recruited between 2023 and 2024 at the National Institute of Neoplastic Diseases (INEN). In the screening cohort, early-stage breast cancer patients were included solely on the basis of stage (0–II). In contrast, the validation cohort included only early-stage patients who had not undergone previous cancer treatment (surgery, chemotherapy, radiotherapy or hormone therapy) or other treatment, in order to avoid potential treatment-induced alterations in miRNA expression profiles.

All participants received detailed information about the study and signed an informed consent form approved by the Institutional Review Board of the INEN, prior to peripheral blood sampling and clinical–pathological data recording. The clinical characteristics of both cohorts are presented in Table 1.

The theoretical sample size was estimated at approximately 64 subjects per group using a power analysis for the comparison of two independent means, assuming a moderate effect size (d = 0.5, Cohen), a significance level of 0.05, and a statistical power of 80%. However, the final sample size included was smaller due to limitations in the availability of biological samples inherent to the clinical setting.

### 2.3. Plasma Collection

Peripheral blood samples were obtained by venipuncture using the Vacutainer system, employing 3 mL polypropylene tubes with EDTA anticoagulant. Samples were transported under controlled temperature conditions (4 °C) within a maximum 3 h after collection. Initial processing for plasma separation was performed in two laboratories, the INEN National Tumor Bank Laboratory of National Institute of Neoplastic Diseases and the Genomics and Integrative Biology Laboratory of the National University of San Marcos (UNMSM). Subsequent analytical processing, including RNA extraction, quantification, and qPCR analysis, was performed entirely at the National Tumoral Bank Laboratory of National Institute of Neoplastic Diseases following the same standardized protocols for all samples.

Blood samples were centrifuged at 2000 rpm for 10 min at 4 °C to separate the plasma. The plasma was then transferred to 1.5 mL tubes and subjected to a second centrifugation at 15,000 rpm for 10 min to remove cell debris. Finally, aliquots (~250 µL) were stored at −80 °C ± 3 °C until analysis. Samples showing visible evidence of hemolysis were recorded during pre-analytical evaluation and excluded prior to RNA extraction and downstream analyses.

### 2.4. miRNA Extraction

miRNA extraction was performed using the miRNeasy Serum/Plasma Kit (Qiagen, Hilden, Germany) following the manufacturer’s recommendations. The procedure is based on lysis with guanidine thiocyanate/phenol, followed by purification on silica columns to obtain nucleic acids free of proteins and other inhibitors.

The concentration and quality of the miRNAs were measured with a NanoDrop 2000/2000c spectrophotometer (Thermo Fisher Scientific, Waltham, MA, USA). The purified miRNAs were stored at −80 °C ± 3 °C until cDNA synthesis.

### 2.5. Reverse Transcription

From the purified miRNAs, the corresponding dilutions were made for cDNA synthesis, using a final concentration of 10 ng in a volume of 7.5 µL of miRNA per reaction. Reverse transcription was performed using the TaqMan MicroRNA Reverse Transcription Kit (Thermo Fisher Scientific Baltics UAB, Vilnius, Lithuania), strictly following the manufacturer’s instructions. The reverse transcription program consisted of: 16 °C for 30 min, 45 °C for 30 min, 85 °C for 5 min and maintenance at 4 °C. For qPCR reactions, 1 µL of cDNA was used.

### 2.6. Quantitative Real-Time PCR (qPCR) Analysis

The quantification of miRNA expression levels in plasma was performed by quantitative real-time PCR (qPCR), following the recommendations of the MIQE guidelines [24] (Supplementary Table S1) to ensure the analytical quality of the experiment. TaqMan™ MicroRNA Assays (Applied Biosystems, Carlsbad, CA, USA, Thermo Fisher Scientific, USA) were used according to the manufacturer’s instructions, using TaqMan™ assays. No-template controls were included during RT-qPCR runs to monitor potential contamination, no AmpErase™ UNG.

Amplifications were performed in the CFX Opus 96 Real-Time PCR System (Bio-Rad, Hercules, CA, USA) under the following program: 50 °C for 2 min, 95 °C for 10 min, followed by 40 cycles at 95 °C for 15 s and 65 °C for 1 min. All reactions were performed in triplicate.

For normalization, the endogenous miR-16 was used, whose stability was evaluated using the NormFinder (version 21; algorithm implemented in Excel) and geNorm (implemented in R version 4.4.3) algorithms, where applicable. Relative expression was calculated using the 2^−ΔΔCt^ method [25]. The sequences of the primers used are available in Supplementary Table S2.

### 2.7. Statistical Analysis

Statistical analyses and graphical representations were performed using GraphPad Prism 10 software (San Diego, CA, USA). Data normality was assessed using the Shapiro–Wilk test. Comparison of the relative expression of miRNAs between groups and their association with clinicopathological variables was performed using Student’s *t*-test for normally distributed variables or the Mann–Whitney test for non-parametrically distributed variables, as appropriate for the data distribution.

A *p*-value < 0.05 was considered statistically significant. Receiver operating characteristic (ROC) curve analyses and Spearman correlations were performed using MedCalc Ltd. (Ostend, Belgium). Binary logistic regression was applied to construct ROC curves for multivariable models combining miRNAs. Model fit and explanatory performance were assessed using the Nagelkerke *R^2^* coefficient for each logistic regression model. To evaluate the stability of model performance estimates, bias-corrected and accelerated (BCa) bootstrap resampling with 1000 iterations was performed for ROC curve analysis and confidence interval estimation. Comparative analyses of individual, reduced, and combined miRNA models were additionally conducted. Youden’s index was used to determine the optimal cut-off point, as well as the corresponding sensitivity and specificity values [26].

## 3. Results

### 3.1. Patient Characteristics

The patients’ ages ranged from 31 to 66 years. In the first phase, 56.3% of breast cancer cases were in women ≤ 51 years of age, with an average age of 44.8 years. In terms of tumor characteristics, 72% had tumors ≤ 2 cm; 68.8% were estrogen receptor-positive and 50.0% were progesterone receptor-positive. The most common molecular subtypes were Luminal A (25.0%) and Luminal B (43.8%).

In the second phase, 50.0% of patients were over 51 years of age, with an average age of 60.7 years. 66.7% reported a family history of cancer, and 83.3% were overweight or obese. Furthermore, 56.7% had tumors ≤ 2 cm; 93.3% and 80.0% were positive for ER and PR, respectively.

The complete characteristics of the patients are detailed in Table 1.

### 3.2. Phase 1: Screening

An initial exploratory cohort was established to analyze the expression of miRNAs such as miR-145, miR-21, miR-210, miR-191, miR-182, miR-335, and miR-125b. This cohort consisted of 10 healthy controlsand 16 patients with early-stage breast cancer, whose clinical information is detailed in Table 1. This screening phase was designed to identify and prioritize candidate miRNAs for further evaluation in a larger validation cohort. Candidate selection was based on a combination of exploratory expression results, preliminary diagnostic performance, and previously reported biological relevance in breast cancer.

#### 3.2.1. Endogenous Stability Analysis

**Figure 2:**
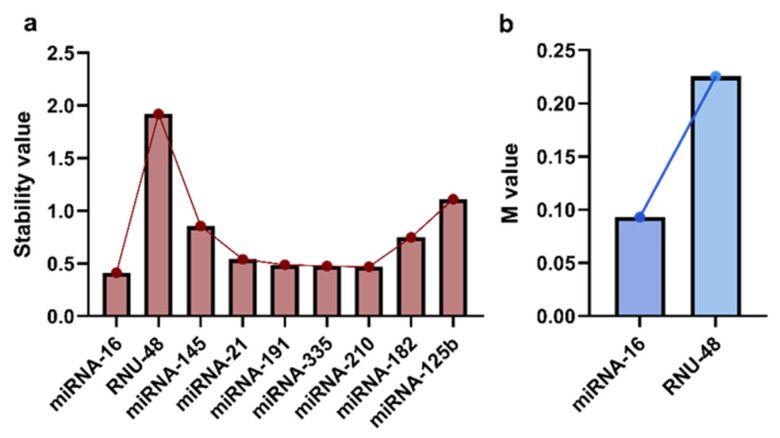
Stability assessment of reference miRNAs. (**a**) NormFinder analysis including endogenous candidate miRNAs (miR-16 and RNU-48) together with miRNAs of interest. (**b**) GeNorm analysis, comparing only miR-16 and RNU-48. Statistical analysis was performed using Student’s *t*-test or the Mann–Whitney U test depending on data distribution and normality.

Previous studies have characterized miR-16 and RNU-48 as endogenous reference miRNAs [27,28]. To validate this selection, their expression stability was evaluated using NormFinder software, obtaining a stability value of 0.212 for miR-16 and 1.972 for RNU-48, compared to the other miRNAs analyzed (Figure 2a).

Additionally, analysis performed with GeNorm software confirmed the suitability of miR-16 as the sole endogenous control, as it had a lower M index (0.09) compared to RNU-48 (0.22) (Figure 2b). These findings support the use of miR-16 as an endogenous reference miRNA (housekeeping gene) in this study, due to its stable expression in all samples analyzed, with a coefficient of variation (CV = 4.0%) compared to RNU-48 (CV = 12.8%).

#### 3.2.2. Expression Levels and Diagnostic Capacity of Candidate miRNAs

**Figure 3:**
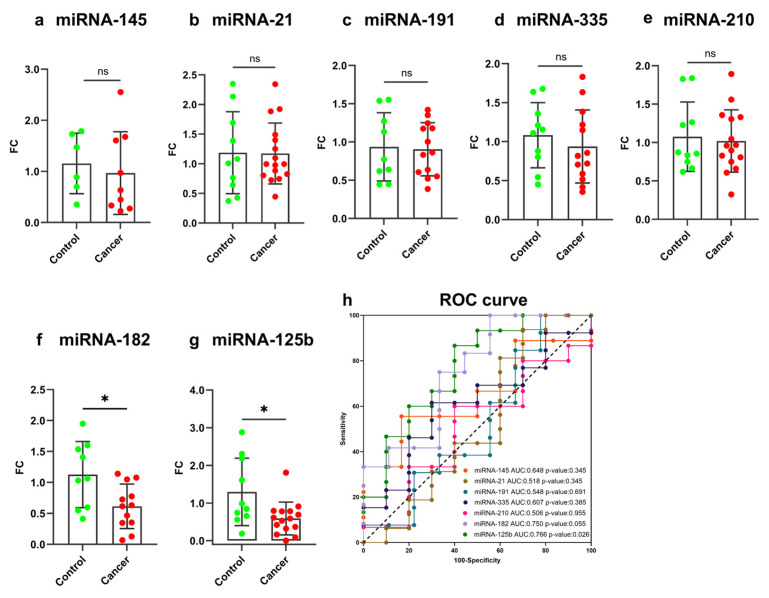
(**a**–**g**) Relative expression of miRNAs in patients with early-stage breast cancer compared to healthy controls. Data are presented as mean ± standard deviation (SD). Statistical analysis was performed using Student’s *t*-test or the Mann–Whitney U test depending on data distribution and normality. (**h**) ROC curves showing the diagnostic performance of each evaluated miRNA. The symbol (*) indicates a significant difference (*p* < 0.05), while “ns” indicates no significant difference. FC: Fold Change.

The variation in the expression of each miRNA was evaluated using the 2^−ΔΔCt^ method. Compared with healthy controls, untreated early-stage breast cancer patients showed lower expression levels of miRNA-182 and miRNA-125b, both of which exhibited statistically significant downregulation. In contrast, miRNA-145, miRNA-21, miRNA-191, miRNA-335, and miRNA-210 did not show statistically significant differences relative to the control group (Figure 3a–g). The average expression values for each miRNA in patients and controls are detailed in Supplementary Table S3.

Among the miRNAs evaluated, miR-182 and miR-125b showed statistically significant differences between cases and controls. In contrast, miR-191 (*p* = 0.658) and miR-335 (*p* = 0.443) did not show statistically significant differences between groups, although both exhibited a downward expression pattern in early-stage breast cancer patients.

Similarly, miR-145, miR-21, and miR-210 showed slight variations (*p* = 0.614, *p* = 0.962, and *p* = 0.978, respectively). A moderate decrease was observed in miR-145 and miR-21, while miR-210 showed a slight relative increase in expression.

The individual performance of miRNAs in discriminating between early-stage breast cancer patients and healthy controls was evaluated using ROC curve analysis (Figure 3h). ROC curve analysis showed that miR-125b had the best discriminatory ability between the two groups, with an area under the curve (AUC) of 0.766 and a *p*-value of 0.026, indicating significant diagnostic potential. In second place, miR-182 achieved an AUC of 0.750 with a *p*-value of 0.055; however, this result did not reach statistical significance.

On the other hand, miR-335 and miR-191 showed AUC values of 0.607 (*p* = 0.385) and 0.548 (*p* = 0.691), respectively. Although neither miRNA reached statistical significance, previous studies have reported their potential biological involvement in breast cancer progression and miRNA deregulation [29,30], respectively, without reaching statistical significance, although both maintained a slight ability to differentiate. In contrast, miR-145, miR-210, and miR-21 showed limited discriminatory capacity and were ruled out as potential biomarkers in this analysis.

The results obtained suggest that miR-182 and miR-125b showed the strongest exploratory diagnostic performance in phase 1, whereas miR-191 and miR-335 were retained for further evaluation based on their expression trends, preliminary diagnostic performance, and previously reported biological relevance in breast cancer.

### 3.3. Phase 2: Validation

Phase 2 was based on combined exploratory selection criteria rather than statistical significance alone.

Based on the evaluation carried out in the first phase, miR-191, miR-335, miR-182, and miR-125b were selected for evaluation in this second cohort. The selection of potential biomarkers was carried out considering 21 healthy controls and 30 untreated early-stage breast cancer patients, whose clinical information is presented in Table 1. The miRNA candidates selected for phase 2 were not chosen based solely on statistical significance at the triage point, but also considering expression trends, preliminary diagnostic performance, and previously described biological relevance in breast cancer.

#### 3.3.1. Expression Levels of Selected miRNAs

**Figure 4:**
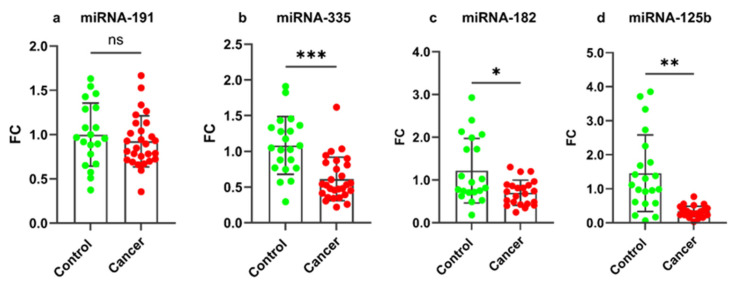
(**a**–**d**) Differential expression of miRNAs in untreated early-stage breast cancer patients compared to healthy controls. Data are presented as mean ± standard deviation (SD). Statistical analysis was performed using Student’s *t*-test or the Mann–Whitney U test depending on data distribution and normality. The symbols (*), (**) and (***) indicate significant differences, corresponding to *p* < 0.05, *p* < 0.01 and *p* < 0.001, respectively, while “ns” indicates no significance. FC: Fold Change.

The differential expression of each miRNA between patients and healthy controls was evaluated using the 2^−ΔΔCt^ method, employing miR-16 as the sole endogenous control. In all cases, untreated patients showed downregulation in the expression of the analyzed miRNAs (Figure 4a–d). The average expression values for each miRNA in patients and controls are detailed in Supplementary Table S4.

The expression of miR-335, miR-182, and miR-125b showed statistically significant differences between the groups. miR-191 showed a non-significant decrease (*p* = 0.439), maintaining a similar trend to that observed in the other downregulated miRNAs.

#### 3.3.2. Expression of miRNAs in Patients According to Clinicopathological Characteristics

**Figure 5:**
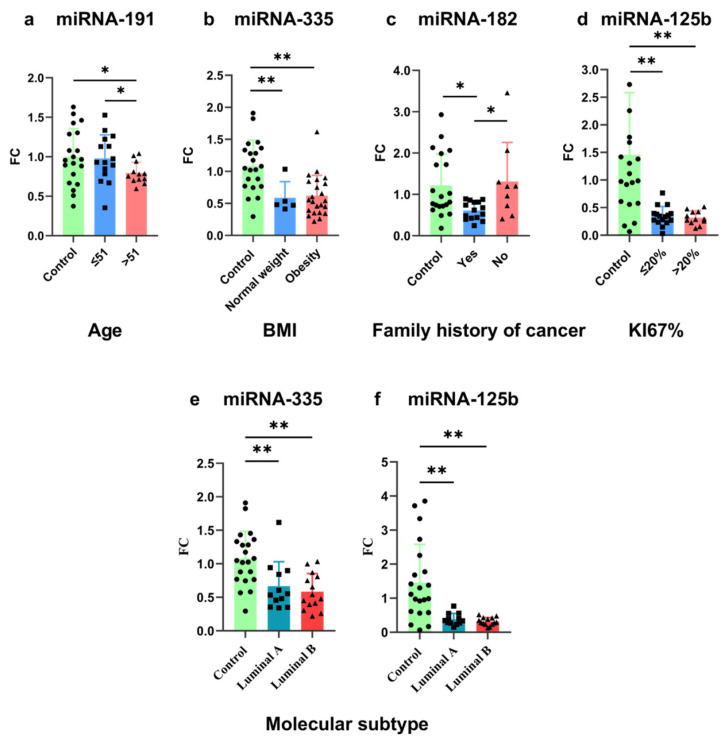
(**a**–**f**) Differential expression of each miRNA evaluated in untreated early-stage breast cancer patients regarding their clinicopathological characteristics. Significance is set at *p* < 0.05 (*), *p* < 0.01 (**) evaluated by *t*-test and Mann–Whitney test depending on data distribution and normality. Data are presented as mean ± standard deviation (SD). FC: Fold Change. Normal weight in BC patients was defined as a BMI of 18.5–24.9 kg/m^2^. Overweight/obesity was defined as BMI ≥ 25 kg/m^2^. Statistical analysis was performed using Student’s *t*-test or the Mann–Whitney U test depending on data distribution and normality.

The differential expression of miRNAs was analyzed according to the clinicopathological characteristics of the patients. miR-191 showed significant differences between subgroups defined by age (*p* < 0.05) (Figure 5a). Likewise, miR-335 showed a significant association with body mass index (BMI) (*p* < 0.01) (Figure 5b), showing a marked reduction in overweight or obese participants. On the other hand, miR-182 showed significant differences in relation to family history of cancer (*p* < 0.05) (Figure 5c).

Regarding the KI-67 proliferative index, miR-125b showed significantly lower expression levels in tumors with higher proliferation (Figure 5d). Finally, both miR-335 and miR-125b showed a significant decrease in Luminal A and Luminal B subtypes compared to healthy controls (*p* < 0.01), suggesting a possible contribution of these miRNAs to specific molecular profiles of breast cancer (Figure 5e,f).

#### 3.3.3. Diagnostic Accuracy of Selected miRNAs

**Figure 6:**
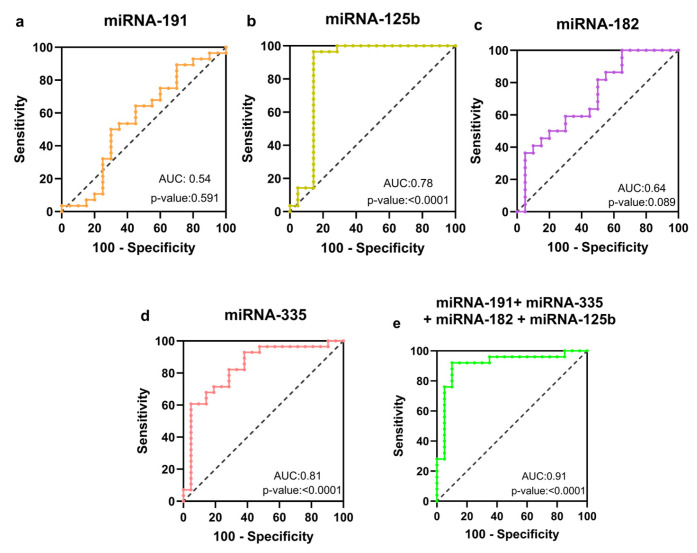
(**a**–**d**) Diagnostic accuracy of miR-191, miR-335, miR-182, miR-125b. (**e**) Combination of miR-191, miR-335, miR-182, miR-125b using logistic regression. ROC curve analysis distinguishes between early-stage breast cancer patients and healthy controls using each miRNA. Statistical analysis was performed using Student’s *t*-test or the Mann–Whitney U test depending on data distribution and normality.

The discriminatory capacity of each miRNA was evaluated using individual ROC curves (Figure 6a–d) The diagnostic performance metrics of the evaluated miRNAs are summarized in Table 2. Among the candidates analyzed, miR-125b and miR-335 showed the best performance, with AUCs of 0.81 (*p* < 0.0001) and 0.78 (*p* < 0.0001), respectively. Spearman’s correlation analysis (Supplementary Table S5) showed a moderate association between miR-125b and miR-335 (ρ = 0.608, *p* < 0.05), supporting their biological consistency as complementary markers. These results are consistent with their marked downregulation in early-stage breast cancer patients, supporting their biological potential as early biomarkers.

To assess whether combining the best individual candidates improved discriminatory performance, multivariable binary logistic regression models were constructed. Initially, a multivariable model integrating miR-125b and miR-335 was developed, showing a moderate improvement in diagnostic performance compared with the individual univariable analyses. The full multivariable panel integrating miR-191, miR-182, miR-335, and miR-125b demonstrated the highest diagnostic performance, achieving an AUC of 0.91 (*p* < 0.0001), with a sensitivity of 92% and a specificity of 90%, indicating a substantial improvement in the overall diagnostic accuracy of the miRNA panel (Figure 6e). Bias-corrected and accelerated (BCa) bootstrap resampling with 1000 iterations yielded a 95% bootstrap confidence interval of 0.776–0.984, providing an internal assessment of the stability and variability of model performance estimates. Furthermore, the four-miRNA model showed the highest Nagelkerke *R^2^* value (0.537) among the evaluated models, indicating superior model fit and explanatory performance compared with the individual and reduced miRNA models. Additional diagnostic performance metrics for all evaluated models are presented in Supplementary Table S5.

Taken together, these findings indicate that although miR-125b and miR-335 showed the best individual performance and their combination provided a moderate improvement, the integration of all four miRNAs into a panel achieved the highest diagnostic performance, providing preliminary evidence for the potential value of combined circulating miRNA biomarkers in early breast cancer detection.

## 4. Discussion

This study provides novel evidence on the diagnostic potential of circulating miRNA panels for early breast cancer detection in a Latin American population, which remains underrepresented in biomarker research. Circulating miRNAs are particularly attractive biomarkers due to their stability in blood and reproducible dysregulation in cancer patients [31].

In the screening phase, seven candidate miRNAs (miR-145, miR-335, miR-210, miR-182, miR-21, miR-125b, and miR-191) associated with tumor processes in breast cancer were quantified. After confirming the stability of miR-16 as an endogenous control, which ensured consistent normalization between samples, their analysis revealed differential expression patterns that provide relevant information about their biological role.

Expression analyses revealed a significant decrease in the expression of miR-182 and miR-125b in patients compared to healthy controls. In the case of miR-182, our findings support its possible involvement beyond the triple-negative subtype, suggesting a cross-sectional role in early stages [32]. On the other hand, our results revealed a significant downregulation of miR-125b, in contrast to the previously reported overexpression in plasma from breast cancer patients by Kassem et al. [33]. This discrepancy could be related to the biphasic behavior of this miRNA [34], whose function may vary depending on the type of sample, tumor subtype, or clinical stage.

miR-191 and miR-335 showed consistent trends toward differential expression, consistent with previous reports on their involvement in tumor aggressiveness [35,36]. Previous reports describe miR-191 as a biomarker with high discriminatory capacity, reaching AUC values close to 0.98 in early stages of breast cancer [37,38]; in our study, its performance was limited. This discrepancy could be attributed to differences in sample type (plasma vs. serum), preanalytical variability, or clinical characteristics of the cohort, such as molecular subtype or tumor stage. However, its tendency toward downregulation is consistent with studies linking it to tumor proliferation processes. In the case of miR-335, although its individual performance was moderate, its expression pattern and functional relevance in pathways associated with tumor progression are consistent with previous findings that propose it as a promising biomarker [39]. This suggests that, although both markers show limitations in performance when used separately, their combined contribution may add value in multivariable models.

In the validation cohort, miR-335, miR-182, and miR-125b remained significantly decreased, while miR-191 showed significant associations with clinicopathological parameters, particularly patient age, reflecting regulation by the tumor microenvironment and hormonal status [40,41]. The downregulation observed for miR-335 is consistent with reports describing its role as a tumor suppressor in both plasma and serum from breast cancer patients [42,43]. This dysregulation has also been documented in tumor tissue, where its decreased expression promotes progression and metastasis, possibly mediated by epigenetic mechanisms [44,45,46]. It has been documented that miR-335 exerts its suppressive action by downregulating key oncogenes such as SOX4, EphA4, and c-Met, inhibiting migration, invasion, and metastasis [47,48,49], as well as modulating apoptosis and proliferation pathways through BRCA1, ER-α, IGF1, Sp1, and ID4 [44].

The downregulation of miR-125b observed in our study contrasts with reports describing its overexpression in the plasma of breast cancer patients, especially in advanced stages and associated with greater tumor aggressiveness and resistance to treatment [50,51,52]. However, in our cohort of untreated early-stage patients, miR-125b showed a significant decrease, which could reflect the loss of its tumor-suppressing functions [53]. This apparently discordant behavior is consistent with the biphasic nature described for miR-125b, which can act as a tumor suppressor by inhibiting genes involved in tumorigenesis, proliferation, invasion, and resistance to apoptosis in early stages [54,55,56,57,58], or as an oncomir in advanced stages of the disease [59,60,61].

Furthermore, miR-125b expression was significantly associated with the Ki-67 proliferation index and with the Luminal A and B molecular subtypes, consistent with studies linking it to tumor behavior characterization and molecular stratification [62,63]. Taken together, these findings reinforce the potential of miR-125b as a relevant component in diagnostic panels for the early detection of breast cancer.

The diagnostic performance was remarkable: miR-125b achieved an AUC of 0.81 with high sensitivity and specificity, and miR-335 showed high specificity (95.24%), suggesting complementary utility in a multimarker panel. When combined with miR-191 and miR-182, the multivariable panel achieved an AUC of 0.91, with a sensitivity of 92% and a specificity of 90%. Importantly, the multivariable model outperformed individual miRNAs, highlighting the added value of integrated biomarker panels for improving diagnostic accuracy in early-stage disease. These findings provide preliminary evidence supporting the potential utility of combined biomarker approaches over single-marker strategies in early-stage breast cancer detection.

However, several limitations should be considered when interpreting these findings. First, the validation cohort was relatively small, which may affect the precision and stability of diagnostic performance estimates. Although internal bootstrap analyses using bias-corrected and accelerated (BCa) resampling with 1000 iterations were incorporated to assess the variability of model performance estimates, these analyses do not replace independent external validation and cannot fully exclude the possibility of model overfitting in small datasets.

Additionally, the single-center design and the absence of external validation cohorts may limit the generalizability of these findings, particularly across geographically and ethnically diverse populations. Although INEN functions as the national referral cancer center receiving patients from multiple regions of Peru, this does not replace independent external validation. Given the unequal access to breast cancer screening and the underrepresentation of Peruvian women in biomarker research, these findings provide initial evidence for this population. Preanalytical variability should also be considered when interpreting the results.

Future multicenter studies including larger and independent external cohorts are necessary to confirm the reproducibility, stability, generalizability, and broader clinical applicability of the proposed miRNA panel.

## 5. Conclusions

miR-125b and miR-335 were significantly dysregulated in early-stage breast cancer and showed promising biological and diagnostic relevance as complementary circulating biomarkers. Their integration with miR-191 and miR-182 into a multivariable liquid biopsy panel was associated with improved diagnostic performance (AUC = 0.91; sensitivity = 92%; specificity = 90%) compared with individual biomarkers. These findings provide initial evidence supporting the potential use of circulating miRNA panels for the early detection of breast cancer, particularly in populations with limited access to conventional screening and those underrepresented in biomarker research. Further validation in larger, independent cohorts is required to confirm the reproducibility, generalizability, and potential clinical utility of these findings.

## Figures and Tables

**Table 1:** Clinical and pathological characteristics of BC patients evaluated in this study.

Characteristics		Phase 1		Phase 2	
		Control (*n* = 10)	Early-Stage BC Patients (*n* = 16)	Control (*n* = 21)	Early-Stage BC ^a^ (*n* = 30)
Age	≤51	6 (60.0%)	9 (56.3%)	10 (47.6%)	15 (50.0%)
	>51	4 (40.0%)	7 (43.7%)	11 (52.4%)	15 (50.0%)
BMI ^b^	Normal weight	3 (33.0%)	7 (43.8%)	7 (33.3%)	5 (16.7%)
	Overweight/Obese	6 (60.0%)	9 (56.2%)	11 (52.4%)	25 (83.3%)
	Unknown	1 (10%)	-	3 (14.3%)	-
Menarche	≤14	7 (70.0%)	13 (81.3%)	17 (80.9%)	24 (80.0%)
	>14	2 (20.0%)	3 (18.7%)	1 (4.8%)	6 (20.0%)
	Unknown	1 (10%)	-	3 (14.3%)	-
Age at first pregnancy	≤20	3 (30.0%)	5 (31.3%)	6 (28.6%)	12 (40.0%)
	>20	7 (70.0%)	11 (68.7%)	12 (57.1%)	18 (60.0%)
	Unknown	-	-	3 (14.3%)	-
Hormonal therapy	Yes	0 (0.0%)	3 (18.8%)	0 (0.0%)	0 (0.0%)
	No	10 (100%)	13 (81.2%)	21 (100%)	30 (100%)
Family history of cancer	Yes	0 (0.0%)	7 (43.7%)	0 (0.0%)	20 (66.7%)
	No	10 (100%)	9 (56.3%)	21 (100%)	10 (33.3%)
Estrogen receptor (ER)	Positive (+)	-	11 (68.8%)	-	28 (93.3%)
	Negative (−)	-	5 (31.2%)	-	2 (6.7%)
Progesterone receptor (PR)	Positive (+)	-	8 (50.0%)	-	24 (80.0%)
	Negative (−)	-	8 (50.0%)	-	6 (20.0%)
Ki-67 index	≤20%	-	8 (50.0%)	-	15 (50.0%)
	>20%	-	8 (50.0%)	-	15 (50.0%)
Tumor size	≤2 cm	-	12 (72.0%)	-	17 (56.7%)
	>2 cm	-	4 (25.0%)	-	12 (40.0%)
	pCR ^c^		-		1 (3.3%)
HER2 status	Positive (+)	-	11 (68.7%)	-	18 (60.0%)
	Negative (−)	-	5 (31.3%)	-	12 (40.0%)
Pathological stage (8th Ed.)	0	-	3 (18.8%)	-	3 (10.0%)
	I	-	7 (43.8%)	-	11 (36.7%)
	II	-	5 (31.4%)	-	16 (53.3%)
Histological grade	1	-	5 (31.3%)	-	3 (10.0%)
	2	-	6 (37.5%)	-	19 (63.4%)
	3	-	6 (31.2%)	-	7 (23.3%)
	pCR		-		1 (3.3%)
Molecular subtype	Luminal A	-	4 (25.0%)	-	13 (43.3%)
	Luminal B	-	7 (43.8%)	-	17 (56.7%)
	HER2	-	2 (12.5%)	-	-
	Triple-Negative	-	3 (18.7%)	-	-

^a^ Breast Cancer without treatment; ^b^ BMI, Body Mass Index; ^c^ pCR, Complete pathological response. Normal weight in BC patients was defined as a BMI of 18.5–24.9 kg/m^2^. Overweight/obesity was defined as BMI ≥ 25 kg/m^2^.

**Table 2:** Diagnostic parameters of each individual and combined miRNA for the early detection of breast cancer using ROC curves, determining the AUC of each miRNA. Youden’s index was calculated to determine the cut-off point, sensitivity, specificity, positive predictive value (PPV), negative predictive value (NPV) and diagnostic accuracy (DA) of each miRNA. SE, standard error.

miRNA	AUC	*p*-Value	SE	95% CI	Youden Index	Cutoff Point	Sensitivity (%)	Specificity (%)	PPV (%)	NPV (%)	DA (%)
miR-191	0.54	0.591	0.086	0.401–0.686	0.18	0.84	46.70%	71.43%	70.00%	48.43%	57%
miR-335	0.78	<0.0001	0.068	0.643–0.885	0.51	0.55	56.67%	95.24%	94.44%	60.63%	73%
miR-182	0.64	0.089	0.083	0.487–0.778	0.27	0.48	32.00%	95.24%	88.87%	54.10%	61%
miR-125b	0.81	<0.0001	0.07	0.684–0.912	0.75	0.55	90.00%	85.71%	89.99%	85.73%	88%
miR-335 + miR-125b	0.79	<0.0001	0.06	0.659–0.895	0.57	0.64	76.67%	80.95%	85.17%	70.86%	78%
miR-191 + miR-335 + miR-182 + miR-125b	0.91	<0.0001	0.05	0.792–0.977	0.82	0.65	92.00%	90.00%	92.00%	90.00%	91%

## Data Availability

All relevant data are included in the manuscript and its Supplementary Materials. No additional datasets were generated or analyzed during the current study.

## Supplementary Materials

The following supporting information can be downloaded at: https://www.mdpi.com/article/10.3390/cancers18121883/s1, Table S1: MIQE 2.0 checklist for authors, Table S2: Mean fold-change expression of evaluated miRNAs in untreated early-stage breast cancer patients relative to healthy controls. Significance values (*p*-values) are shown. Table S3: Mean fold-change expression of each evaluated miRNA in untreated early-stage breast cancer patients relative to healthy controls. Table S4: Spearman correlation coefficients between each pair of miRNAs. * indicates a significant correlation coefficient (*p*-value < 0.05). Table S5: Comparative ROC performance and internal bootstrap confidence intervals of individual, reduced, and combined miRNA models.
