# Supplementary material for: Circulating Free miRNAs as Liquid Biopsy Biomarkers for Early Detection of Breast Cancer in Peruvian Women
Source: Cancers (Basel). 2026 Jun 9;18(12):1883. doi: 10.3390/cancers18121883 (PMC13297568; doi:10.3390/cancers18121883)
Supplement: Supplementary file 1 [file cancers-18-01883-s001.zip › cancers-4298190-supplementary.pdf]

Table S1. MIQE 2.0 checklist for authors.

	PROVIDED[a]	DESCRIPTION/JUSTIFICATION[b]
**1. REAGENT PREPARATION**		
Bioinformatics tools and versions and settings used to design assays	NO	Thermo probes / Materials and Methods
Official gene symbol, species and sequence accession number	YES	Thermo probes / Materials and Methods
Location of amplicon	YES	Thermo probes / Materials and Methods
Amplicon length	YES	Thermo probes / Materials and Methods
Primer and probe sequences[c]	YES	Thermo probes / Materials and Methods
Location and identity of any modifications	NO	Not applicable
Manufacturer of oligonucleotides	YES	Thermo Fisher Scientific Baltics UAB, Lithuania/ Materials and Methods
Details of optimization performed	NO	Not applicable
**2. SAMPLE PREPARATION**		
Detailed description of sample types and numbers	YES	Cohorts evaluated from plasma / Materials and Methods
Sampling procedure (including time to storage)	YES	Transport at 4 °C, <3 hours / Materials and Methods
Sample aliquoting, storage conditions and duration	YES	–80 °C, 250 µL aliquots / Materials and Methods
Description of extraction method including amount of sample processed	YES	miRNeasy Kit (Qiagen), following the manufacturer's protocol / Materials and Methods
Source and amount of spike-in nucleic acids added	NO	Not applicable
Volume of elution buffer used to elute/resuspend nucleic acids	YES	14 µL of RNase-free water, NanoDrop / Materials and Methods
Number of extraction replicates	NO	Not applicable
Extraction blanks and percent yield included	NO	Not applicable
Method to evaluate quality and quantity of nucleic acids	YES	Evaluated with a NanoDrop 2000/2000c (Thermo Fisher) / Materials and Methods
Storage conditions: temperature, concentration, duration, buffer, aliquots	YES	–80 °C ± 3 °C until cDNA synthesis / Materials and Methods
Clear description of dilution steps used to prepare working template solution	YES	10 ng/ Materials and Methods
Template modification (digestion, sonication, pre-amplification, DNAse treatment etc.)	NO	Not applicable
Purification after modification	NO	Not applicable
**3. REVERSE TRANSCRIPTION**[d]		
cDNA priming method and primer concentration	YES	kit TaqMan MicroRNA Reverse Transcription Kit (Thermo Fisher Scientific Baltics UAB, Lithuania)/Materials and Methods

One or two-step protocol (include reaction details for two-step)	YES	One-step / Materials and Methods
Amount of RNA used per reaction	YES	7.5 µL of miRNA / Materials and Methods
Detailed reaction components and conditions	YES	TaqMan MicroRNA Reverse Transcription Kit (Thermo Fisher Scientific Baltics UAB, Lithuania) / Materials and Methods
Estimated copies measured with and without addition of RT[e]	NO	Not applicable
Manufacturer of reagents, catalog number and lot number	YES	TaqMan MicroRNA Reverse Transcription Kit (Thermo Fisher Scientific Baltics UAB, Lithuania) / Materials and Methods
Storage of cDNA: temperature, concentration/dilution, duration, buffer, aliquots	YES	At –20 °C in aliquots / Materials and Methods
**4. qPCR PROTOCOL**		
Template treatment (initial heating or chemical denaturation)	YES	Denaturation at 50 °C / Materials and Methods
Primer and probe concentration in the reaction and source	YES	TaqMan MicroRNA Assays probes (Applied Biosystems, Thermo Fisher Scientific, USA) / Materials and Methods
Polymerase identity and concentration, Mg^{2+} and dNTP concentrations[f]	YES	TaqMan™ Universal PCR Master Mix, no AmpErase™ UNG / Materials and Methods
Buffer/kit (manufacturer, catalog number and lot number)	YES	TaqMan™ Universal PCR Master Mix, no AmpErase™ UNG / Materials and Methods
Complete thermocycling parameters including reaction volume	YES	qPCR programming / Materials and Methods
Manufacturer and type of qPCR instrument	YES	CFX Opus 96 Dx (Bio-Rad, USA) / Materials and Methods
**5. DATA ANALYSIS**		
Storage and submission of raw fluorescence data using RDES[g] or RDML[h]	YES	Exported from Bio-Rad software / Materials and Methods
Identity of standards (synthetic, plasmid, genomic, IVT[i], mRNA etc.) and method of quantification	NO	Not applicable
Method of baseline correction and Cq determination	YES	Software Bio-Rad/ Materials and Methods
qPCR analysis program (source, version)	YES	Software Bio-Rad/ Materials and Methods
Details of positive and negative controls	NO	
Frequency and Cq of negative controls	NO	
Examples of positive and negative results	NO	
PCR efficiency estimation and method for its determination	NO	

Method of target quantity calculation	YES	2^-ΔΔCt method / Materials and Methods
Description of replicates	YES	Triplicates / Materials and Methods
Repeatability (intra-experiment variation)	YES	%CV / Materials and Methods
Reproducibility (inter-experiment/user/lab etc. variation)	YES	Variability of miRNAs in patients and healthy participants / Materials and Methods
Limit of detection calculated?	NO	Not applicable
Dynamic range (limits of quantification)	NO	Not applicable
Method of validation of reference genes	YES	miR-16 validated with NormFinder/GeNorm / Materials and Methods
Description of normalization method / calculation of normalized expression	YES	Normalized with miR-16 / Materials and Methods
Statistical methods used for analysis	YES	Parametric and non-parametric tests / Materials and Methods
Choice of significance level and calculation of statistical power	YES	$p < 0.05$ / Materials and Methods
Specificity (when measuring rare mutations, pathogen sequences etc.)	YES	High, using TaqMan probes / Materials and Methods

Table S2. Mean fold-change expression of evaluated miRNAs in untreated early-stage breast cancer patients relative to healthy controls. Significance values (*p*-values) are shown.

miRNA identification	Gene expression			
	Control[a]	BC[b]		
	FC[c] (Mean ± SD)	FC[c] (Mean ± SD)	Reg.	p-value
miRNA-145	1.155 ± 0.592	0.968 ± 0.809	↓	0.614
miRNA-21	1.185 ± 0.691	1.174 ± 0.514	↓	0.962
miRNA-191	1.317 ± 1.277	1.016 ± 0.392	↓	0.658
miRNA-335	1.082 ± 0.417	0.937 ± 0.468	↓	0.443
miRNA-210	1.075 ± 0.451	1.162 ± 0.691	↑	0.978
miRNA-182	1.127 ± 0.534	0.614 ± 0.358	↓	*0.026
miRNA-125b	1.296 ± 0.894	0.957 ± 1.525	↓	*0.039

[a]Control healthy subjects; [b]Breast Cancer patients; [c]Fold Change ($2^{-\Delta\Delta Ct}$)

Table S3. Mean fold-change expression of each evaluated miRNA in untreated early-stage breast cancer patients relative to healthy controls.

miRNA identification	Gene expression			
	Control[a]	BC[b]		
	FC[c] (Mean ± SD)	FC[c] (Mean ± SD)	Reg.	p-value
miRNA-191	1.138 ± 0.724	1.016 ± 0.453	↓	0.439
miRNA-335	1.082± 0.403	0.715 ± 0.486	↓	**<0.0001
miRNA-182	1.217 ± 0.755	0.983± 0.859	↓	*0.020
miRNA-125b	1.457 ± 1.126	0.583 ± 1.073	↓	*0.0002

[a]Control healthy subjects; [b]Breast cancer patients; [c]Fold change ($2^{-\Delta\Delta Ct}$)

Table S4. Spearman correlation coefficients between each pair of miRNAs. * indicates a significant correlation coefficient (*p*-value < 0.05).

	miRNA-191	miRNA-335	miRNA-182	miRNA-125b
miRNA-191	1	*0.547	0.262	*0.329
miRNA-335		1	*0.330	*0.608
miRNA-182			1	0.164
miRNA-125b				1

Table S5. Comparative ROC performance and internal bootstrap confidence intervals of individual, reduced, and combined miRNA models.

Model	Biomarkers included	AUC	95% CI / Bootstrap BCa 95% CI	Nagelkerke R^2
Individual	miR-335	0.78	0.614-0.884	0.184
Individual	miR-125b	0.81	0.627-0.929	0.192
Reduced panel	miR-335 + miR-125b	0.79	0.648-0.912	0.267
Full panel	miR-191 + miR-182 + miR-335 + miR-125b	0.91	0.776–0.984	0.537
